# Modeling the Role of Cancer-Associated Fibroblasts in Tumor Cell Invasion

**DOI:** 10.3390/cancers14040962

**Published:** 2022-02-15

**Authors:** Stephanie Poon, Laurie E. Ailles

**Affiliations:** 1 Department of Medical Biophysics, University of Toronto, Toronto, ON M5G 1L7, Canada; stephaniee.poon@mail.utoronto.ca; 2 Princess Margaret Cancer Centre, University Health Network, Toronto, ON M5G 1L7, Canada

**Keywords:** cancer-associated fibroblast (CAF), tumor invasion, tumor microenvironment (TME), invasion assay, in vitro modeling, tumor modeling

## Abstract

**Simple Summary:**

Accurate in vitro modeling of diseases is essential to making breakthrough and clinically relevant discoveries. Assays to examine the process of invasion—a classical hallmark of cancer—have evolved over the years to overcome shortfalls in their design and accommodate new knowledge in the field, such as the role of the tumor microenvironment (TME) in propagating this process. The goals of this review are two-fold: To walk through the tried-and-true plus novel and new invasion assays currently used in cancer research with a focus on those incorporating cancer-associated fibroblasts (CAFs), and to be a resource for researchers to find the correct invasion assays that suit their own unique needs and biological questions.

**Abstract:**

The major cause of cancer-related deaths can be attributed to the metastatic spread of tumor cells—a dynamic and complex multi-step process beginning with tumor cells acquiring an invasive phenotype to allow them to travel through the blood and lymphatic vessels to ultimately seed at a secondary site. Over the years, various in vitro models have been used to characterize specific steps in the cascade to collectively begin providing a clearer picture of the puzzle of metastasis. With the discovery of the TME’s supporting role in activating tumor cell invasion and metastasis, these models have evolved in parallel to accommodate features of the TME and to observe its interactions with tumor cells. In particular, CAFs that reside in reactive tumor stroma have been shown to play a substantial pro-invasive role through their matrix-modifying functions; accordingly, this warranted further investigation with the development and use of invasion assays that could include these stromal cells. This review explores the growing toolbox of assays used to study tumor cell invasion, from the simple beginnings of a tumor cell and extracellular matrix set-up to the advent of models that aim to more closely recapitulate the interplay between tumor cells, CAFs and the extracellular matrix. These models will prove to be invaluable tools to help tease out the intricacies of tumor cell invasion.

## 1. Introduction

Cancer continues to be a leading cause of mortality worldwide, with the World Health Organization reporting nearly 10 million deaths attributed to the disease in 2020 [1]. Despite the plethora of cancer types and their varying rates of lethality, the overarching primary cause of cancer-related deaths is attributed to metastasis: A dynamic multi-step process where tumor cells from the primary lesion ultimately seed themselves at a distant site in the body to spawn secondary malignant lesions. Metastasis involves a large number of molecular and phenotypic changes in the cell that must occur to facilitate this process [2]. Briefly, a cell first needs to acquire a migratory phenotype allowing them to invade the adjacent stromal tissue; studies have shown that cells undergo a process called the epithelial-to-mesenchymal transition (EMT) in the initial steps of metastasis as it augments cellular motility [3,4,5]. Cells then intravasate into nearby blood or lymphatic vessels and must survive the journey through the vascular system before extravasating out into the distant site. Cells that make it out must then survive in the microenvironment of the new site and restart their proliferative programming to successfully colonize the new tissue. Studies have demonstrated that these cells at the metastatic site express epithelial markers such as E-cadherin, suggesting they have gone through the reverse process of the mesenchymal-to-epithelial transition (MET) in this last step of the cascade. Altogether, this forms what is known as the EMT/MET model of metastatic dissemination [6,7]. This model in itself continues to be challenged and updated with new experimental evidence; while both are important, EMT and MET may not be necessary nor sufficient for the onset of metastasis, and the extent of their contribution varies widely with disease context. Additionally, the idea that EMT and MET are binary all-or-nothing processes has changed into more of a spectral view, where metastasizing cells can be in a partial state of either [7,8,9]. Evidently, cells face a seemingly impossible uphill battle to the end point, but the few hardy cells that make it often lead to the demise of their host patient.

To say metastasis is complex is almost an understatement and unraveling all the biological intricacies of metastasis has been an ongoing endeavor for decades. A starting point for the hunt for therapeutic targets to slow down or halt this cascade is to pin down the genes and molecular pathways responsible for the multitude of changes occurring during the metastatic process [10,11]. A sensible approach to this undertaking is not dissimilar to solving a complicated puzzle: Breaking it down into smaller pieces and tackling each one separately. Correspondingly, the foundational principles of metastasis were first established by dissecting the metastatic cascade into physiological stages and deriving laboratory models to study each stage in more detail. With this plan of attack, in vitro modeling proved useful in exploring one of the earliest yet most pivotal stages of metastasis: The onset of stromal invasion. This consists of the ability of a cancer cell to detach and migrate away from the primary lesion or towards a vessel, and the physical and biochemical modifications of the surrounding extracellular matrix (ECM; the network of macromolecules such as collagens, proteoglycans and glycoproteins that support the parenchymal cells in the tissue) to allow the cancer cell to do so. Recent studies have demonstrated that neighboring cells in the stroma, particularly cancer-associated fibroblasts (CAFs), play key roles in these requirements for tumor cells to invade. This review will focus on the assays and models used to study the vital process of invasion in metastasis and, more recently, the important role that CAFs play to augment it.

## 2. Early Modeling of Cell Migration and Invasion

Unsurprisingly, the earliest invasion assays were derivatives of pre-existing migration assays: Assays that observe the rate and extent of cell movement on a two-dimensional plane. Cell migration in itself is not exclusive to the context of cancer; processes such as embryonic morphogenesis and immune cell trafficking rely heavily on cells migrating to the correct locations to perform their function [12]. One of the earliest and most widely used in vitro methods to evaluate cell migration is the transwell assay system, developed by Dr. Stephen Boyden in 1961 and often coined the “Boyden chamber” system [13]. The classical set-up involves upper and lower chambers separated by a porous membrane. Commercially available transwell systems consist of an upper well insert with the membrane at the bottom. Cells of interest are seeded on top of the membrane, and the insert is placed into the well of a multi-well plate (commonly a 24-well plate) containing media. Cells that migrate through to the other side of the membrane in response to the contents of the media can then be stained and quantified. This assay is well suited to evaluate migration driven by chemotactic gradients [14]. Another popular migration assay is the scratch or wound-healing assay, where a cell-free area (the “scratch”) is made in a confluent monolayer of cells, and the rate at which this area closes through cell migration is evaluated [15,16]. A near-identical set-up can be achieved by creating an exclusion zone: Situating a “plug” or insert that creates a physical barrier on the plastic surface before seeding cells around it. After the cell layer is established, the barrier is removed, creating a cell-free area on an unaltered surface that cells can migrate into [17,18,19]. These are the preferred methods to evaluate collective or sheet migration in an established cell layer; unlike the Boyden Chamber system, cells are given time to establish physiologically relevant cell–cell contacts prior to inducing migration [20]. However, chemotactic gradients are much more difficult to evaluate in this assay; thus, both types of migration assays have a unique place within the toolbox of in vitro migration assays. 

**Figure 1:**
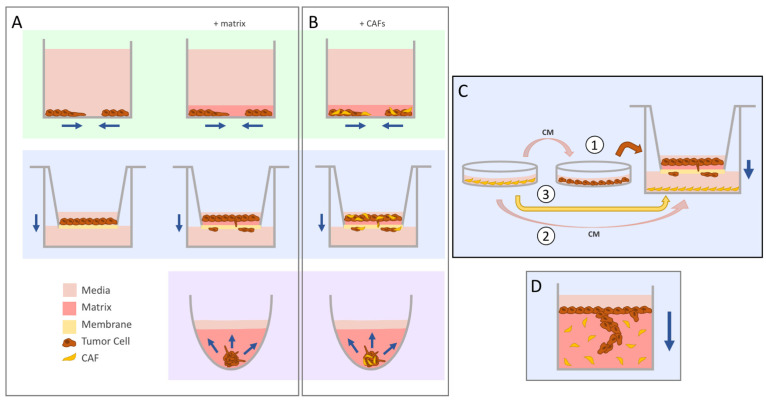
The evolution of migration into invasion assays. Navy arrows indicate the direction of movement by the tumor cells. (**A**) The scratch wound or exclusion zone (green, top) and transwell (blue, middle) assays were commonly used to study migration in vitro. The addition of a matrix for cells to travel through allowed for the seamless conversion of these systems to study invasion instead. Concerns regarding the translatability of 2D cyto-architecture prompted the use of 3D spheroids (purple, bottom), which can be embedded in matrix to evaluate invasion of cells coming out from the aggregate. (**B**) Cancer-associated fibroblasts (CAFs) can be added into these systems by seeding the cells as a co-culture mix. (**C**) There are different ways to study CAF-influenced tumor cell invasion in the transwell set-up. CAF-conditioned media (CM) can be used to pre-treat tumor cells prior to seeding into the insert (1) or added directly to the lower chamber (2). CAFs can also be seeded directly into the bottom chamber (3). (**D**) The vertical gel assay yields high-resolution images of invading cells through sectioning and staining.

What figuratively and literally separates invasion from migration is the presence of a matrix; while cell migration is a requirement for invading cells, the reverse is not true [14]. Conveniently, the aforementioned assays can be modified to incorporate a matrix into their set-up, allowing researchers to easily study both biological processes without investing in extra equipment (Figure 1A, top and middle row). In the transwell system, the porous membrane is coated with a layer of the matrix before seeding cells on top [21], whereas in both the scratch wound and exclusion zone systems, a layer of the matrix is added to embed the cells after the cell-free area has been established in the monolayer [22]. In both cases, collagen or preparation of a basement membrane matrix such as Matrigel (Corning) or Cultrex (R&D) are the most commonly used.

One of the limitations of these particular set-ups is that they do not mimic the physiological state of cells found in the body. Because of their spatial arrangement, cells cultured in a monolayer on a plastic dish are not subjected to the same chemical and oxygen gradients, and also differ in their types of cellular contacts [23,24]. Three-dimensional in vitro models were developed to address these drawbacks, the most common being the use of spheroids or cell aggregates. Under low-attachment conditions, some cell lines have the ability to self-aggregate into a stable, transferrable cluster or “spheroid” of cells. These structures intrinsically already have a more relevant “cyto-architecture” than 2D monolayers with nutrient and drug uptake, proliferation and intercellular signaling [25]. When comparing 2D and 3D culture systems, substantial changes in gene expression are seen, which can significantly affect downstream results and conclusions [26,27,28]. The invasion of cells from the spheroid can be assessed by embedding the spheroid in the matrix (Figure 1A, bottom) and measuring the outward invasion of cells over time [29]. As more time-efficient methods for generating large numbers of spheroids were developed, such as the use of microwell-containing multi-well plates (e.g., AggreWell™ plates), microfluidics-based or bioreactor-based methods [30], spheroid invasion assays have gained traction as a popular method to gauge the invasive potential of cells of interest; however, not all cell lines have the ability to self-aggregate into spheroids, and therefore the feasibility of this assay is dependent on this particular feature.

## 3. CAFs and the Tumor Microenvironment

It is now well established that cancer pathogenesis is not single-handedly driven by cell-intrinsic mutations. In solid tumors, early reductionist views of a cancerous tumor as a homogenous unit have evolved into what can be seen as an organ system containing a heterogeneous population of malignant cells, supported by the surrounding stromal tissues and non-malignant cell populations that co-evolve with the tumor mass and contribute to its progression [31]. The tumor microenvironment (TME) encompasses the non-malignant cellular and non-cellular components within and around the tumor. A number of cell types enter, reside and leave this space throughout the course of a tumor’s growth. A variety of immune cells of both the innate and adaptive response can populate the TME, and both the type of cell and their location relative to the tumor affect patient prognosis. For example, while tumors infiltrated with cytotoxic CD8^+^ T-cells generally correlate with a positive patient prognosis, the opposite is true for tumors high in regulatory T-cell numbers. Macrophages in the TME tend to be polarized into the M2 anti-inflammatory, pro-tumorigenic subtype versus the M1 pro-inflammatory subtype, and other cells such as neutrophils and dendritic cells can suppress or promote tumor growth depending on the stage of the tumor. [32,33] Tumor endothelial cells lining blood vasculature in the TME generally form more leaky vessels (where tumor cells can more easily pass through) and can also release factors to create an environment more suitable for tumor cells to metastasize [34]. Additionally, the cellular composition of the TME varies between tumor locations within the body. Stellate cells—a mesenchymal precursor cell—are found in the liver and pancreas, while adipocytes are critical cell types in the realm of breast cancer [35].

In many solid tumors, often the most prominent cellular component is made up of stromal fibroblasts. Fibroblasts are vital players in maintaining tissue homeostasis, and under normal physiological conditions these spindle-shaped cells are quiescent but readily activated by stimuli in their environment. They are the main workhorses in the synthesis of ECM proteins such as collagens, laminins and fibronectin. They also have full reign over ECM re-modeling and turnover by having the ability to secrete matrix metalloproteinases (MMPs), which degrade ECM proteins [36,37], and lysyl oxidases, which cross-link collagen and elastin and increase the stiffness of the matrix [38,39]. These play a role in the acute wound healing response—a process where the presence of functional fibroblasts is absolutely essential [40]. The state of the stroma in both a freshly healing wound and a tumor share many similarities: Inflammation is present, and ECM is being produced and re-modelled [40]. Accordingly, a tumor can be seen as a “wound that never heals” [41]. In the wound healing response, activated fibroblasts are often labelled as myofibroblasts due to their increased intrinsic contractility necessary to heal wounded tissue [42]. In the context of cancer, these cells are given the term cancer- or carcinoma-associated fibroblasts [36,43]. CAFs contribute in several ways to the classic hallmarks of cancer [44,45], but their role in promoting tumor cell invasion is particularly interesting due to the clear relevance and connection between fibroblast function and the requirement for ECM remodeling to permit tumor cells to move through. In line with this notion, early in vitro models of invasion were adjusted in order to study tumor cell invasion in a CAF-inclusive system. This includes not only another set of upgrades for pre-existing models, but also the advent of completely novel set-ups and methods of analyses to accurately interpret the output of said experiments. While the same need for upgrades can be expanded to models for all the other stages of metastases, this review will focus specifically on in vitro invasion assays that incorporate CAFs into their set-up, and the breakthrough findings as a result of these assays.

## 4. Incorporation of CAFs into Pre-Existing Models

### 4.1. Scratch Wound/Exclusion Zone Invasion Assay

The invasion assays discussed so far are, for the most part, amendable to the inclusion of a second cell type. For the scratch wound or exclusion zone set-ups for collective cell invasion, CAFs can be incorporated into the cell monolayer prior to creating the cell-free area and adding the matrix (Figure 1B, top). Using a live-cell imaging system with compatible cell culture plates, Neri et al. saw that the inclusion of patient-derived CAFs enhanced cancer cell invasion. By labelling their lung cancer cell line with a fluorescent red nuclear protein, they could monitor CAF and cancer cell invasion separately. Additionally, generating clones from the bulk population of CAFs revealed that individual CAFs generated from the same patient tumor promoted varying levels of invasion [46].

### 4.2. Transwell Invasion Assay

Despite limitations of the transwell system, it is still a popular and widely used method to evaluate both migration and invasion, mainly due to its accessibility (inserts are commercially available), its relative ease of set-up, and to an extent, its versatility to measure two features of aggressive cancer cell behavior (Figure 1C). For example, CAFs can either be mixed with cancer cells and laid on top of the matrix-coated membranes (Figure 1B, middle), or cancer cells can be pre-treated with CAF-conditioned media (CAF-CM) before being seeded into the inserts [47,48]. To model CAF-guided invasion, CAFs can be grown or CAF-CM can be added to the bottom chamber below the insert [49,50]. A study by Sun et al. cleverly used the transwell set-up as a means to co-culture their CAFs and oral cancer cells; exosomes and cytokines secreted by the CAFs could pass through the porous membrane without the two cells physically interacting with each other. They then isolated exosomes from CAF-CM, added them to the culturing media of their cells and saw that the exosomes specifically enhanced tumor cell invasion [51].

On occasion, higher-resolution imaging of this dynamic process is desired. This can be achieved with a set-up that allows for downstream staining and imaging. An example of this is the vertical gel invasion assay (Figure 1D), which functions similarly to transwell assays in that the direction of invasion is downwards into the matrix. This type of set-up has been in use for several decades [52], and the crux of the assay involves the preparation of an ECM gel (CAFs can be incorporated into the gel), allowing it to set in the desired thickness and shape, and seeding cells of interest on top. Once the overlaid cells have had time to invade, the gels can be fixed, sectioned and stained to visualize the depth of cancer cell invasion into the matrix [53]. In an elaborate study by Gaggioli et al., they found that CAFs created tracks within the matrix (a mixture of Matrigel with collagen type I), which squamous cell carcinoma (SCC) cells were able to travel through. They incubated matrix blocks with embedded CAFs for several days and removed all the CAFs before seeding cancer cells on top. Despite the absence of CAFs, the tracks they had previously created remained in the matrix, and cell invasion was still observed. Their study ultimately revealed that CAF-led collective cell migration of SCC cells was dependent upon Rho-ROCK function, as well as integrin-α3 and -α5 expression specifically in the CAFs [54]. This assay was also used by the same group to characterize the signaling pathways leading to an “epigenetic switch” of fibroblasts in the TME into a pro-invasive phenotype. In this specific study, they identified the inhibition of DNMT1 and JAK signaling as a candidate strategy to suppress CAF-associated pro-invasive activity [55].

### 4.3. Spheroid Invasion Assay

The spheroid invasion assay is advantageous when cell–cell contacts and cell–matrix contacts are of interest. CAFs can be incorporated into spheroids to generate co-culture spheroids ready for matrix embedding (Figure 1B, bottom). This is particularly useful with cancer cell lines that are unable to self-aggregate, as CAFs on their own will form tight spheroid structures and can help facilitate the generation of a spheroid in co-culture [56]. CAFs can also be seeded into the surrounding matrix of monoculture spheres (similar to the vertical gel set-up) to model the scenario of a tumor mass surrounded by CAF-populated stroma. A study by Attieh et al. looked at the invasion of tumor cell spheroids embedded in a dome of collagen with or without CAFs. They also evaluated, through the use of CAF-CM and the presence of CAFs around the exterior of the collagen dome, whether the CAF secretome was sufficient to drive a more robust invasion. Their experiments support previous findings in the literature that CAF-secreted factors alone are not sufficient to drive invasion, and that matrix remodeling by CAFs within the matrix enhances tumor cell invasion. Furthermore, they identified fibronectin deposited by CAFs as an MMP-independent enhancer of tumor cell invasion [57]. Another advantage of the spheroid invasion assay is its capability to be scaled up to allow for multiple conditions to be set up within a single experiment. For example, Mei et al. used 96-well, low-attachment, U-bottom plates, which allow for the generation of spheroids and the addition of a matrix to occur within the same plate. With this set-up, they investigated the protective effects of CAFs in co-culture spheroids on radiation and how it affected the CAF-mediated invasion of breast cancer cells [56]. To supplement the use of round-bottom plates, live-imaging systems (e.g., the Sartorius Incucyte^®^) now have image-acquisition programs optimized for the imaging of round bottom plates together with in-house analyses of the invasive area from spheroids, allowing for a more automated and seamless workflow [29].

Another method of visualizing co-culture spheroids is by embedding them in a drop of the matrix on top of a glass-bottom cell culture dish to perform higher-resolution fluorescence imaging such as confocal microscopy. By tagging CAFs and tumor cells in different-colored fluorescence markers, high-magnification images are able to image CAFs, leading a trail of cancer cells away from the main spheroid body as demonstrated by Conti et al. [58]. Finally, co-culture spheroids can also be layered on top of a bed of matrix to evaluate cells moving away from the main spheroid body. Although, strictly speaking, this model does not assess the matrix invasion, it evaluates cell migration on a more biologically relevant substrate compared to conventional techniques using cell culture plastic. As with the studies described above, invasive strands can be seen radiating away from the spheroid body led by CAFs using this method [59,60]. These studies showed that once CAFs and cancer cells made contact, the two cells remained in close contact with CAFs and cancer cells in a leader–follower organization even in the absence of ECM. Furthermore, CAFs exerted pulling forces on cancer cells via E-cadherin/N-cadherin junctions, which were required for collective cancer cell invasion [59].

## 5. The Rise of Novel In Vitro Models

As our understanding of the importance of the TME in cancer progression continued to grow, it became evident that in order for in vitro models to recapitulate a tumor’s pathobiology as accurately as possible, its interactions with the TME must be considered. Improvements to modeling the main tumor mass have extended to making improvements on how to also model the cellular and non-cellular components of the TME that are relevant to the biological questions being asked. Some cancers have unique features exclusive to their pathobiology; pancreatic ductal adenocarcinoma (PDAC) tumors, for example, are known for having extremely stiff matrices and sub-populations of CAFs that contribute in different ways to the overall pathobiology of the disease [61,62].

### 5.1. Matrix Modifications

A component where improvements can be made is the matrix itself. Invasion patterns of the same cell line can differ between types of matrices [63], so the choice of the matrix to study invasion is important depending on the desired biological context. The majority of studies settle with the widely available murine basement membrane extract (Matrigel, Cultrex) or rat-tail collagen; these can also act as a suitable starting base to add additional ECM elements such as fibronectin. However, batch effects, particularly in Matrigel, can be an unwanted source of variability between experiments. One option explored by Scherzer et al. is the generation of fibroblast-derived ECM. Fibroblasts cultured on a coated dish will continuously secrete matrix proteins, and upon confluency, the formed matrix can be decellularized and used. They generated the CAF matrix on the underside of the porous membrane situated at the bottom of a transwell insert and demonstrated that the matrix acted as a chemoattractant to lung cancer cells seeded on the other side of the membrane. Furthermore, cancer cells seeded on top of the matrix exhibited higher velocity migration versus cells seeded on the fibronectin-coated controls [64]. Similar results were seen in a study by Lee et al., where they derived acellular matrices from normal pancreatic stellate cells (PSCs, the predominant stromal cell type in the pancreas) and PDAC-associated fibroblasts (PDAC-AFs). They also generated a mouse fibroblast line (NIH-3T3) overexpressing the gene for fibroblast activation protein (*Fap*), a classical marker of CAFs. They found that matrices generated by FAP^−^ and FAP^+^ CAFs closely resembled those from PSCs and PDAC-AFs, respectively, and that an invasive PDAC cell line migrated faster and further on the matrix generated by PDAC-AFs [65]. Finally, the use of a mouse-derived mesenteric tissue was explored by Ghose et al. as the matrix of choice for tumor cells to invade through via a modified transwell set-up. The mesentery is a sheet of connective tissue that connects the intestine to the anterior wall of the peritoneal cavity and has a composition close to the basement membrane. Using mesentery tissue harvested from mice allows for a physiological recapitulation closer to an in vivo system but comes with the heavier cost of needing to sacrifice mice to obtain the tissue [66]. Overall, although these methods are potentially more physiologically relevant, a major drawback is that their integration into pre-existing invasion assays is limited to those that can incorporate a solid piece of the matrix, as the matrix is not in a liquid state and thus unable to be pipetted.

### 5.2. Novel Alternative Invasion Assays

A small but steady stream of novel alternative invasion models has entered the literature over the years, varying in both ease of set-up and complexity. Aslan et al. recently described a simple 3D invasion model where a single-cell suspension is mixed with Matrigel and seeded as a droplet in a multi-well plate. Invasive cells will move through the droplet and migrate outwards onto the plastic, which can be imaged daily with a standard brightfield microscope. These cells can also be fixed and stained with antibodies for proteins of interest at the endpoint, providing additional information within the same experiment [67]. This sets it apart from previously discussed invasion assays in that invasion can be captured over a time course, and an end-point analysis can be performed on the same system of cells and matrix. Although CAFs were not incorporated in their model, it would be interesting to see how this assay would perform if CAFs were mixed into the matrix drop along with tumor cells. Zhang et al. devised a “dumbbell” model of invasion, where a droplet of Matrigel containing CAFs was connected to a separate drop of Matrigel containing cancer cells via a “causeway” or connecting block of Matrigel. Interestingly, they found that CAFs within their droplet self-assembled into a large network of long structures. This expanded through the causeway into cancer cell territory, where they interacted and engulfed growing clusters of cancer cells. Closer inspection of the invasive front showed cancer cells adapting to the network of CAFs and directionally migrating along it [68].

### 5.3. Using Organoids in Invasion Assays

While the use of cancer cell lines comes with their ease of growing and amenability for genetic modifications, the tradeoff is their limited translatability when it comes to interpatient tumor heterogeneity within the same type of cancer. This can be addressed with the use of organoid models: Self-organizing 3D organotypic structures derived from stem/progenitor cells embedded in the matrix and cultured in conditions mimicking the appropriate stem cell niche. The discovery of organoid cultures was groundbreaking for many different diseases, including cancer [69,70]. Organoid models hold several advantages, including the retention of features of the source organ such as intestinal crypts in colorectal or ductal structures in pancreatic organoids. Organoids can be generated from both healthy and tumor tissue, making them a favorable model of choice in the realm of drug development and personalized medicine; several groups have generated biobanks of matched healthy and tumor patient-derived organoids with characterized mutational status [71,72,73]. Patient tumor-derived organoids have been shown in several different cancer types to hold histopathological similarities to the original tumor [74]. Unfortunately, not all cancer types are able to generate organoid cultures, making this type of culture selective towards these few diseases. However, organoids can be derived from select murine cell lines harboring specific mutations or transgenes, making them extremely useful for more biological mechanistic studies such as pathway characterization [75]. Kasashima et al. generated mouse tumor organoids (MTOs) from a mouse model of a particular phenotype of colorectal cancer and co-cultured them with colonic fibroblasts (CFs) in a 3D culture system. They noted that CFs with a knockout mutation in the *Prkcz* gene have classical CAF characteristics [76]. Hanley et al. also observed CAF-induced invasion in a murine organoid model of breast cancer, using specific mouse lines that spontaneously develop mammary tumors closely resembling human breast cancers. In their study, they also isolated the stromal cell population from their bulk tumors through the depletion of other cell types (immune, epithelial, erythroid), resulting in matched pairs of organoids and stromal cells. In this co-culture system, they showed that cancer cells released TGFβ, which induced NOX4 expression in stromal cells. This resulted in matrix remodeling, and correspondingly NOX4 inhibition reduced invasion [77].

## 6. Compartmentalizing the Tumor

**Figure 2:**
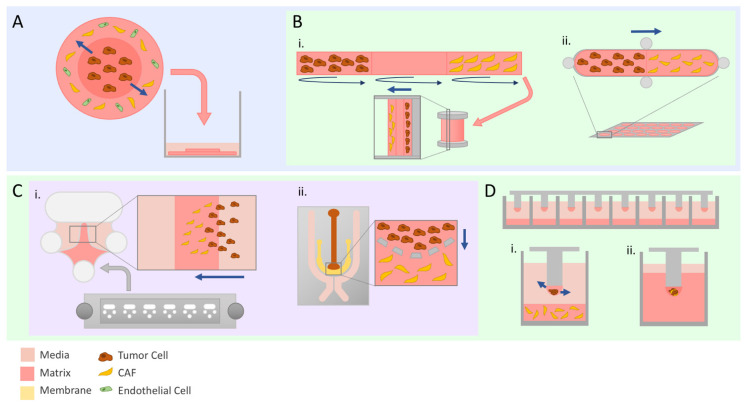
Advanced modeling of tumor invasion. Straight navy arrows indicate direction of tumor cell invasion. Schematics illustrate set-ups used by discussed studies. (**A**) The tumoroid model is composed of two matrix discs representing tumor and stroma synthesized separately and then assembled into one entity before being submerged in media. (**B**) Bioengineered models with customizable compartments. TRACER (i) is a 3D model where cells are seeded on marked sections of a biocomposite strip before being rolled to replicate physiologically relevant hypoxia and nutrient gradients. GLAnCE (ii) has two compartments and is suited specifically for looking at the interface between the two. (**C**) Microfabricated chips are a cost-effective way to set up invasion assays due to the small amounts of material required. The Iuvo Invasion Assay slide (i) contains two side ports and a center port where matrix can be deposited. Microfluidics chips (ii) can be particularly useful to evaluate the effect of chemokine or cytokine gradients due to the design of the media channels on the outer portion of the chip surrounding the cell-containing inner channels. (**D**) Micropillar chips allow for scaling up of invasion experiments. Tumor cells and CAFs can either be seeded separately to evaluate paracrine interactions (i) or a co-culture spheroid can be embedded in the droplet (ii) prior to being fully embedded.

CAF interplay is far more complex than the interaction between CAFs and tumor cells. There remained a need to have better control over modeling the various components of the stroma, with the flexibility to include or exclude certain aspects of the TME. A solution came in the form of the “tumoroid” model. Tumoroids are systems where the central cancer “mass” and its surrounding matrix are created separately, before coalescing the two into one unified system (Figure 2A). The “stromal” element of the system can include one or a combination of different types of stromal cells embedded in a matrix of choice. In a tumoroid model of colorectal cancer, a central mass of HCT29 cells was shown to invade into the stromal unit; this unit was composed of collagen with or without the addition of laminin, the endothelial cell line HUVEC and human dermal fibroblasts (HDFs) [78,79]. Pape et al. took this model one step further and set up a series of tumoroid models: One with an acellular stromal component, one containing HUVEC and HDFs and a third containing patient-derived CAFs. They observed a more robust invasion of their cancer line and interestingly reduced vascular network formation with the CAF-embedded stroma compared to the other two tumoroids [80].

Within the repertoire of invasion assays, models that could evaluate other parameters such as gene expression or proliferative potential (i.e., high-content experiments) at the experimental endpoint were relatively lacking. Evaluating CAF–tumor cell interactions with more precision often requires higher-optical-resolution imaging such as confocal microscopy, or extensive processing of the sample at the endpoint. Scientists became creative in the realm of bioengineering to develop platforms to address these pitfalls in the pool of available assays. For example, the Tissue Roll for Analysis of Cellular Environment and Response (TRACER) model mimics the natural nutrient and oxygen gradients present in a tumor mass by seeding cells on a biocomposite strip and rolling it around a central spool (Figure 2B(i)). At the end of the experiment, the strips are unrolled, and cells can be processed and analyzed in various ways. Perhaps the most appealing feature of TRACER is the ability to quickly harvest cells from different locations in the strip to perform downstream experiments with the knowledge of what spatial region the collected cells originated from [81]. This model was adapted in a proof-of-concept study by Dean et al. to demonstrate its utility in evaluating CAF-mediated tumor cell invasion using head and neck squamous cell carcinoma cell lines. The strip was divided into three regions (each representing one full wrap around the spindle), and two different co-culture methods were tested: Where CAFs and tumor cells were seeded on opposite ends of the strip, and where CAFs and tumor cells were mixed into the same single region. The strip’s remaining regions were laid with collagen, before being rolled around the spindle and placed into a well of culture media. They were able to show that the presence of CAFs in either set-up promoted tumor cell invasion radially through the layers independent of proliferative effects. Furthermore, they demonstrated that the concentration of collagen used in each region affected the rate of tumor cell invasion seen, making this an extremely versatile platform for generating the desired conditions [82,83]. Another platform that focuses on the interface between CAFs and tumor cells is Gels for Live Analysis of Compartmentalized Environments (GLAnCE), developed by D’Arcangelo et al. This consists of a custom plate containing 24 channel-like pill-shaped structures with ports at the ends where cells in a collagen suspension could be injected. A gelation step between the injection of cell types at either end of the channel allows for distinct compartments with a precise interface to be generated (Figure 2B(ii)). This plate of channels is compatible with standard widefield fluorescence imaging, and similarly to TRACER, cells can be harvested from the collagen slabs after imaging for further downstream experiments. In their study, images of fluorescently labelled cells showed morphological changes suggestive of an invasive phenotype in tumor cells at the CAF–tumor interface, as well as increased migration outwards from the interface [84].

## 7. Engineering a Dynamic Tumor

TRACER and GLAnCE are two examples of models that went beyond the limits of standard lab plasticware. The freedom to bioengineer a platform of interest opened doors to not only design more intricate and physiologically accurate in vitro models, but also to tackle practical considerations or limitations that pre-existing invasion assays have.

### 7.1. Making It Small

Microfabrication models are scaled down such that they require a lot less biological material for setting up an experiment. This feature makes them an attractive option for studying primary cell types that are difficult to harvest or expand. They also can be designed to accommodate the desired imaging modality; for example, thinner chips can allow antibodies used for staining to penetrate the sample through a matrix or be adaptable for live cell imaging [85,86]. The Beebe lab developed LumeNEXT, a small device consisting of a chamber with a rod resting across the center; the rod size and shape can be selected by the user to generate the desired luminal structures [87]. A matrix of choice is added to the chamber to encapsulate the rod, and the rod is removed after matrix gelation resulting in a cylindrical space—the lumen structure—through the matrix. Lugo-Cintrón et al. used this device to investigate breast cancer invasion from a luminal space into the surrounding CAF-embedded matrix. The versatility of the device was highlighted, as they harvested conditioned media from the device at wells located on the ends of the cancer cell-filled lumen chamber as well as demonstrating its compatibility with second harmonic generation and fluorescent imaging modalities [88]. Another microdevice called the Iuvo 3D Invasion slide (Figure 2C(i)) was used by Rai et al. to evaluate the fibroblast-led invasion of cancer cells orchestrated specifically from cancer cell-derived exosomes. Exosome-treated fibroblasts were allowed to transmigrate into the center chamber of Matrigel first, before the addition of fluorescently-labelled cancer cells. While neither exosomes alone nor vehicle control-treated fibroblasts initiated cancer cell invasion, cancer cells were seen trailing behind exosome-treated fibroblasts into the matrix [89].

Under this same umbrella, microfluidics models—devices containing micro-channels to hold fluid—were used to study invasion based on the notion that tumors and their microenvironment constantly change throughout the course of disease progression. Under stressors such as hypoxia, nutrient deprivation or drug treatments, tumor cells are capable of adapting to these selective pressures. A system where certain parameters can be more precisely controlled or modified over time can provide extremely relevant information regarding how those changes affect cellular responses [90]. A microfluidics chip developed by Truong et al. has adjacently placed channels where cancer cells and matrix can be injected separately, allowing for cell–matrix interactions at the channel interface (Figure 2C(ii)). Similar to the tumoroid models, their chip allows for the customizable compartmentalization of the tumor and stroma but has the additional feature of being able to generate chemoattractant gradients through the use of surrounding media channels that feed into the cells [91]. The addition of CAFs into the stromal matrix compartment of their chip enhanced the invasion of breast cancer cell lines relative to a matrix-only control. Additionally, CAF–tumor cell interactions could be imaged in real time, and RNA sequencing could subsequently be performed on tumor cells extracted and sorted out of their chip co-culture system, showcasing the utility of their device [92].

### 7.2. Expanding the Utility of Small Devices

The appeal of high-throughput and high content assays has led to the development of several innovative platforms. The Kuh lab has developed a mini-pillar chip where multicellular spheroids are generated on the tips of each pillar before being inverted over a 96-well plate filled with culture media (Figure 2D). This platform is unique in that spheroids can be subjected to culture conditions and treatments of interest, and then while remaining on the same pillar chip, be embedded either in Optical Cutting Temperature (OCT) compound for cryo-sectioning or Histogel to be processed into a paraffin block for sectioning and staining [93,94,95]. They further applied this model by seeding a layer of collagen embedded with PSCs at the bottom of the wells (Figure 2D(i)). PDAC spheroids embedded in collagen solution were generated on the pillars and, after gelation, placed above the PSCs to allow for paracrine signaling between the two cell types. With the convenience of performing both optical and mechanical sectioning, they showed that PDAC spheroids co-cultured with PSCs had the accelerated presence of invadopodia and a remodeled ECM richer in type I collagen and fibronectin. Furthermore, PDAC spheroids were collected from the pillars after several days of incubation, and through Western Blot protein analysis were shown to upregulate the expression of EMT-related proteins [96]. In a similar fashion, Puls et al. created a system where a series of small posts are spaced apart on a sheet such that each post will be three-dimensionally centered in a well of a 96-well plate. A small droplet of cancer cells suspended in a self-assembling oligomeric collagen mixture (Oligomer) is pipetted on the tips of each small post and allowed to polymerize. The wells of a 96-well plate are filled with Oligomer before inverting the plate over the posts to encapsulate the droplet in the matrix (Figure 2D(ii)). The posts attached to the sheet can then be removed, suspending the cell–matrix sphere in the center of the matrix. Because the tumor cells are first encapsulated in a small droplet before being embedded, they were able to generate tumor droplets using a mixture of patient-derived PDAC and CAF cells and demonstrated that CAFs induced more PDAC cell invasion relative to PDAC-alone spheres. The practicality of their system was also demonstrated through a drug screen coupled with an automated confocal imaging system [97].

### 7.3. Observing Matrix Changes

While the contents of the surrounding tumor stroma are important considerations, a contributing factor that is often overlooked is the stiffness of the matrix itself. CAFs have a direct influence on matrix stiffness through the cycling of pre-existing matrix protein degradation and new matrix protein deposition, as well as the degree of cross-linkage of ECM fibers. Experimentally, stiffness can be altered through the concentration of polymer, the water content and the amount of cross-linking between fibers. Modifying the concentration of polymer affects the pore size of the mesh of proteins, impacting the ability of cells to travel through it; meanwhile, cross-linking fibers, through processes such as nonenzymatic glycation, affect stiffness without changing fiber density. A systematic review conducted by Micalet et al. on the effect of physical microenvironment on tumor cell invasion found a positive correlation between stiffness and the extent of invasion in the range of stiffnesses that are comparable to values found in in vivo studies [98]. Throughout the course of the invasion, CAFs present in the TME can affect the state of the ECM. To investigate this, Saini et al. developed what they call an “open-top” 3D tumor-stroma model, where a sheet of CAF-embedded hydrogel in a holder is stamped with small well shapes. After polymerization of the gel, cancer cells are seeded into these wells and allowed to attach before submerging the system in cell media [99]. Apart from being able to image invading cancer cells from the microwells in real time, the exposed surface allowed for biophysical measurements to be made using atomic force microscopy and confocal reflectance microscopy. These measure stiffness and collagen fiber density, respectively, and through their system they quantitatively showed that a matrix embedded with CAFs becomes progressively stiffer and more collagen-dense over time [100].

## 8. Conclusions and Current Perspectives

The study of cell invasion has flourished over the years with both the continued use of tried-and-true techniques and the advent of innovative models. The latter has been driven in part by the widely accepted notion that the CAF population residing in the TME can influence the rate and extent of solid tumor cell invasion and could no longer be ignored in conventional modeling. This review highlights the journey of invasion assay development as design limitations were acknowledged and addressed, while pre-existing assays were adapted to accommodate desired features of physiological tumors. In parallel, this has pushed similar degrees of advancement in our knowledge and understanding of the invasion process, bringing to light the microenvironmental factors that contribute to it. The mechanisms by which CAFs promote tumor cell invasion in a wide range of tumor types are numerous, from typical cell–cell signaling to dynamically changing the ECM—a consequence of fibroblast remodeling activity. With the ever-expanding toolbox of assays, care must be taken to select the assays that best address the scientific questions being asked. They should be able to accommodate the specific biology of the tumor type being studied, while being compatible with the right downstream applications. Table 1 compiles important features of consideration in the mentioned assays. In general, there exist tradeoffs between experimental practicality, complexity and the availability of resources to set up the assay. Despite the handful of known limitations of the transwell assay system, it continues to be the assay of choice for many standard wet labs due to its availability and ease of use. While many of the bioengineered models steer closer to being more physiologically relevant, their use by other groups will depend on whether the physical components of the system can be synthesized in bulk and distributed. They also tend to be more labor-intensive, with many not being adaptable for high-throughput use. In the meantime, using a combination of several models continues to be an ideal compromise for covering the limitations of a singular assay.

As laboratory and engineering technologies continue to improve, many of the more intricate assays aim to be a stepping-stone to in vivo modeling, which is generally more physiologically relevant at the cost of longer experimental durations, lower throughput and a higher price tag. CAFs are advantageous in that they are one of the few cell types that are straightforward to culture out from resected tumors and maintain; the inclusion of additional patient-derived cells or ECM coupled with parallel analyses of patient clinical data are powerful traits to have for an in vitro model that can better guide relevant follow-up in vivo experiments. Another take-home point is that some of these models contain multiple compartments or channels, giving them the flexibility to include other stromal cell types such as endothelial or immune cells. As a hypothetical example, in tumors where the infiltration of certain immune populations is commonly seen, these models can aid in evaluating the contribution of each cell type to a given phenotype. Furthermore, there are ongoing efforts to develop in vitro models of metastasis, a process commonly studied using mouse models. While not covered in this review, it is worth mentioning that efforts in microfluidics particularly have been ongoing to create systems that can study particular features such as the metastatic dormancy of cells [101]. An elaborate “plug-and-play” set-up by Ni et al. allows for the study of the metastatic cascade in its entirety [102].

In summary, acknowledging the body of evidence that CAFs play essential roles in tumor cell invasion has led to remarkable advances in both reinventing old and creating new in vitro models of tumor cell invasion. Invasion assays have solidified their place in both basic and translational research and will continue to be a key piece in assembling the puzzle of the metastatic cascade. Perhaps the key to halting metastasis lies in targeting the first step of the cascade, making studying the process of invasion and its key players even more vital. Proper in vitro models will pave the way towards relevant in vivo experiments and promising clinical studies, hopefully leading to the ultimate goal of preventing metastasis, the primary cause of cancer-related deaths.

## Figures and Tables

**Table 1:** Summary of all the assays along with key experimental features.

Assay	Ref.	Type	Technical Complexity	Material Availability ^1^	HT Potential ^2^	Compatible Imaging Techniques ^3,4^	Quantitative Measurement(s) ^4^	Location of CAFs in Assay ^4^	Cell Collection from Assay
Scratch Wound	[15,16]	Timecourse	Low	Common + Commercial	Yes	Live Cell (PC, Fluorescence)	Speed of gap closure	Mixed with tumor cells	Yes
Exclusion Zone	[17,18,19]	Timecourse	Low	Commercial	Yes	Live Cell (PC, Fluorescence)	Speed of gap closure	Mixed with tumor cells	Yes
Transwell	[13,21]	Endpoint	Low	Commercial	No	Crystal Violet Staining	# of cells on membrane	See Figure 1C	No
Vertical Gel	[53]	Endpoint	Moderate	Somewhat Specialized	No	H&E Staining of sections	Invasive Index ^5^	In matrix separated from tumor cells	No
Spheroid	[29]	Timecourse	Low	Common	Yes	Live cell (PC, Fluorescence)	Invasive Area, Circularity	Mixed with tumor cells; Can be in surrounding matrix	Yes
Matrigel Drop	[67]	Both	Low	Common	No	Live cell (PC, Fluorescence), IF staining	Invasive Area (Migrating edge)	Can be in surrounding matrix	No
3D Dumbbell	[68]	Timecourse	Moderate	Common	No	Live cell (PC, Fluorescence)	N/A	In matrix separated from tumor cells	No
Organoids	[76,77]	Both	Moderate	Somewhat Specialized	Yes	Live cell (PC, Fluorescence), IF staining, H&E staining of sections	Organoid features (Number, size, circularity)	In surrounding matrix	Yes
Tumoroid	[78,79]	Endpoint	Moderate	Commercial	No	IF staining, Optical Projection Tomography	Invasion features (Distance, Area, Aggregate size)	In stromal compartment	Yes
TRACER	[81]	Endpoint	High	Specialized	No	IF staining, Scanning Electron Microscopy	Proportion of tumor cells in layer of interest	In matrix separated or mixed with tumor cells	Yes
GLAnCE	[84]	Timecourse	Moderate	Specialized	No	Live Cell (Fluorescence)	Interface features (# of strand structures, Aggregate circularity)	In matrix separated or mixed with tumor cells	Yes
LumeNEXT Chip	[87]	Timecourse	High	Specialized	No	Live cell (Fluorescence), Second Harmonic Generation	Cell migration distance, # of migration cells	In surrounding matrix	No
Iuvo Invasion Slide	[89]	Timecourse	High	Commercial	No	Live Cell (Fluorescence)	# of invading cells	Added to same or opposite port from tumor cells (See Figure 2C(i))	No
Microfluidics Chip	[91]	Both	High	Specialized	No	Live cell (PC, Fluorescence), IF staining	Invasive cell features (Distance, Number, Speed)	In matrix separated from tumor cells	Yes
Mini-Pillar	[93,94,95]	Both	High	Specialized	Yes	Live cell (Fluorescence), IF staining of whole sample or sections	Length and # of protrusions, Circularity and # of spheroids	In matrix separated from tumor cells	Yes
HT-HC Platform	[97]	Endpoint	High	Specialized	Yes	IF staining	Invasive cell features (Distance, Number)	Mixed with tumor cells	No
Open-top Model	[99]	Both	Moderate	Specialized	No	Live cell (PC, Fluorescence), Real time cell tracking, IF staining, Confocal reflectance microscopy	Area disorder ^5^, Migration Index ^5^, Speed of Migration	In surrounding matrix	Yes

^1^ Common = can be set up with standard lab plasticware, Commercial = full kit or specialized parts can be purchased, Specialized = not commercially available, made in-house; ^2^ HT = High throughput. Gauged as ability for assay to be scaled up to the equivalent of a 96-well plate and/or be automated regarding set-up and data acquisition; ^3^ PC = Phase contrast, H&E = Hematoxylin and Eosin, IF = Immunofluorescence; ^4^ Demonstrated in mentioned studies, but are not limited to those listed; ^5^ Formula or calculation for metric described in referenced paper.
